# Immunotherapy – 2074. Tolerability of sublingual immunotherapy with tropical mite allergen vaccines using different dosing schedules in asthmatic children

**DOI:** 10.1186/1939-4551-6-S1-P156

**Published:** 2013-04-23

**Authors:** Raúl Lázaro Castro Almarales, Mirta Alvarez Castello, José Severino Rodríguez Canosa, Mercedes Ronquillo Díaz, Mayda González León, Mary Carmen Reyes Zamora, Maytee Mateo Morejó

**Affiliations:** 1 Allergens Department, National Center of Bioproducts (BIOCEN), Allergology and General Integral Medicine, La Habana, Cuba; 2 Allergology Department, University Hospital General Calixto García, Havana, Cuba; WAO Member; Cuban Society of Allergy, Asthma and Clinical Immunology, Member; Cuban Society of Immunology Member; 3 Allergen Service, University Hospital "General Calixto García", Havana, Cuba; WAO Member; Cuban Society of Allergy, Asthma and Clinical Immunology, Member; Cuban Society of Immunology Member; 4 Allergen Service, University Hospital "General Calixto García", Havana, Cuba; 5 Docent Polyclinic "Pedro Fonseca", Havana, Cuba Cuban Society of Integral General Medicine, Member; Cuban Society of Allergy, Asthma and Clinical Immunology, Member; Cuban Society of Immunology Member; 6 Allergen, Biocen, Cuba

## Background

Immunotherapy with mite allergens is effective both in rhinitis and asthma. However, injection immunotherapy carries a high risk of systemic adverse events, so, the sublingual route (SLIT) is being used more frequently. Efficacy of SLIT in asthma has been widely studied, however, in Cuba, the therapeutic effect of mite vaccines of *Dermatophagoides pteronyssinus* (Dp), *D.siboney* (Ds) and *Blomia tropicalis*(Bt) has been demonstrated only in adults, but not children. This aim is to determinate adherence and tolerability of SLIT with mite allergen vaccines using different treatment schedules in asthmatic children.

## Methods

One hundred and twenty children (2 to 15 years) with mild to moderate asthma were selected for each treatment schedule. Three dosing schedules were used: (1) maximum of 3 daily drops; (2): max. 5 drops and (3) max. 10 drops, all by sublingual route. Incremental updosing was used for 3 weeks. A maintenance dose of 2000 BU was used once a week for schedule 1 and twice a week for schedules 2 and 3, during 2 months. Standardized House Dust Mite allergen vaccines of *Dermatophagoides pteronyssinus*, *D. siboney* and *Blomia tropicalis*were employed (VALERGEN, BIOCEN, Cuba).

## Results

Adherence to treatment was above 90% for the three schemes with no statistically significant difference (p>0,08) among them. The patients tolerated the different dosing schedules without having to change the planned scheme. Only two patients reported local adverse reactions, classified both as grade I (mild tongue itching without medication).

## Conclusions

Summarizing, all children tolerated the three schedules similarly and adherence was high.

